# Maternal amino acid metabolites during pregnancy and preterm birth: results from two prospective cohort studies

**DOI:** 10.1186/s12916-026-04710-5

**Published:** 2026-02-18

**Authors:** Jingnan Chen, Yidan Dong, Jiajun Zhao, Yuwei Lai, Congmei Xiao, Yuanqing Fu, Ke Zhang, Meng Ye, Wanglong Gou, Shijia Hu, Zelei Miao, Fan Li, Ping Wu, Tianlei Wang, Jiaying Yuan, Yayi Hu, Jin Wu, An Pan, Xiong-Fei Pan, Ju-Sheng Zheng

**Affiliations:** 1 Affiliated Hangzhou First People’s Hospital, School of Medicine, Westlake University, Hangzhou, Zhejiang China; 2 Westlake Laboratory of Life Sciences and Biomedicine, Hangzhou, China; 3 Section of Epidemiology and Population Health & Department of Gynecology and Obstetrics, Ministry of Education Key Laboratory of Birth Defects and Related Diseases of Women and Children & Children’s Medicine Key Laboratory of Sichuan Province, West China Second University Hospital, Sichuan University, Chengdu, China; 4 Department of Epidemiology and Biostatistics, Ministry of Education Key Laboratory of Environment and Health, School of Public Health, Tongji Medical College, Huazhong University of Science and Technology, Wuhan, 430030 China; 5 Department of Science and Education, Shuangliu Maternal and Child Health, Chengdu, China; 6 Department of Obstetrics and Gynecology, Ministry of Education Key Laboratory of Birth Defects and Related Diseases of Women and Children, West China Second University Hospital, Sichuan University, Chengdu, China; 7 Department of Pediatrics, Ministry of Education Key Laboratory of Birth Defects and Related Diseases of Women and Children, West China Second University Hospital, Sichuan University, Chengdu, Sichuan 610041 China; 8 West China Biomedical Big Data Center, West China Hospital, Sichuan University, Chengdu, China; 9 Shuangliu Institute of Women’s and Children’s Health, Shuangliu Maternal and Child Health Hospital, Chengdu, 610041 China; 10 School of Future Biomedicine and School of Life Sciences, Westlake University, Hangzhou, China; 11 Zhejiang Key Laboratory of Multi-Omics in Infection and Immunity, School of Medicine, Westlake University, No. 600 Dunyu Road, Sandun Town, Hangzhou, China

**Keywords:** Maternal amino acid metabolites, Preterm birth, Gestational duration, Biomarker, Birth cohort

## Abstract

**Background:**

Precise regulation of amino acid concentrations in the maternal–fetal circulation is essential for fetal development, but the association of maternal amino acid metabolites with gestational duration and preterm birth risk remains obscure. Therefore, we aimed to identify biomarkers consistently associated with gestational duration and preterm birth risk across cohorts.

**Methods:**

The discovery cohort is a nested case–control study based on the Tongji-Huaxi-Shuangliu Birth Cohort (THSBC), which recruited pregnant women in early pregnancy (6–15 weeks of gestation) between 2017 and 2020 and followed the women during middle and late pregnancy periods. In this discovery cohort we performed trajectory analysis for the amino acid metabolism related metabolites across 3 pregnancy periods. Regression modeling was performed to identify amino acid metabolites that were associated with gestational duration and preterm birth. We also developed an amino acid preterm prediction score (APPS) based on the identified metabolites to stratify patients according to their risk of preterm birth. Validation of the identified metabolites and the APPS was performed in the Westlake Precision Birth Cohort study (WeBirth) consisting of pregnant women with gestational diabetes recruited in mid pregnancy (22–28 weeks’ gestation) between 2019 and 2023.

**Results:**

A total of 723 participants (241 preterm cases) in the THSBC and 1597 participants (96 preterm cases) in the WeBirth were included. In the THSBC, each 1 standard deviation increase in Ile-Val in mid pregnancy was associated with an increment of 0.19 (95% CI, 0.03, 0.35) week for gestational duration, while Val-Gly was inversely associated with gestational duration (− 0.20; 95% CI, − 0.34, − 0.06). In addition, lower Ile-Val and higher Val-Gly were associated with higher risk of preterm birth (OR, 0.72; 95% CI, 0.57, 0.91; OR, 1.25; 95% CI, 1.03, 1.51, respectively). These associations were consistent in the WeBirth cohort. Compared to the clinical model, the incorporation of APPS exhibited better performance in predicting preterm birth.

**Conclusions:**

The findings suggest that circulating amino acid metabolites may serve as biomarkers for preterm birth prediction. Maternal amino acid metabolites may have potential clinical utility in improving prenatal risk assessment.

**Supplementary Information:**

The online version contains supplementary material available at 10.1186/s12916-026-04710-5.

## Background

Preterm birth, a leading cause of death in children under 5 years old, poses a severe threat to global neonatal health [1–3]. Current clinical biomarkers for preterm birth prediction have limitations like the need to perform invasive measurements to assess transvaginal cervical length and insufficient sensitivity in low-risk nulliparous women [4]. Omics strategies (e.g., vaginal and plasma metabolome, extracellular vesicles lipidome, and cell-free RNA sequencing) facilitate the discovery of novel putative biomarkers with predictive potential for preterm birth [5–10], while their reproducibility has not yet been consistently demonstrated. The identification of replicable preterm birth biomarkers for clinical translation remains challenging.

Amino acids are core substrates in the maternal–fetal nutrient transport, but evidence about the relationships of circulating amino acids and related metabolites with gestational duration and preterm birth remain sparse. Precise regulation of amino acid concentrations in the maternal–fetal circulation is essential for fetal development [11], with several prior studies linking specific amino acids to fetal development [12–14]. In addition, women with intrauterine growth restriction have higher plasma concentrations of several essential amino acids than non-affected pregnant women [15]. Similarly, maternal circulating amino acid levels are shown to closely correlate with birth weight [16]. However, the role of other amino acids and related metabolites during pregnancy and preterm birth is still unclear.

Therefore, in the present study, we aimed to investigate the temporal dynamics of maternal amino acid metabolism throughout pregnancy, and to identify potential amino acid biomarkers associated with gestational duration and preterm birth among pregnant women. As exploratory analyses, we investigated the potential determinants (included demographic characteristics, clinical traits, diet and lifestyles, maternal genetics, and gut microbiota) of the above identified key amino acid metabolites.

## Methods

### Study population

Our study was mainly based on the Tongji-Huaxi-Shuangliu Birth Cohort (THSBC), which recruited women aged 18–41 years during their visits on the Shuangliu Maternal and Child Health Hospital for routine antenatal examination in early pregnancy [17]. The exclusion criteria have been described previously [17], which included receiving fertility treatment, reporting severe chronic or infectious diseases, or non-completion of questionnaires at the baseline visit. A total of 7281 pregnant women were recruited between 2017 and 2020. In each pregnancy period (early pregnancy, 6 + 0–15 + 6; mid pregnancy, 16 + 0–28 + 0; and late pregnancy, > 29 + 0 weeks + days of gestation), the participants were interviewed by trained staff and completed questionnaires. Maternal blood and fecal samples were also collected in each pregnancy period. The THSBC study was approved by the Ethics Committee of Tongji Medical College, Huazhong University of Science and Technology (Number: [2017]-(S225)−1). Written informed consent was obtained from all participants.

For the present study, we established a nested case–control study within the THSBC and included a total of 723 participants consisting of all the participants delivering preterm (*N* = 241) and age-matched controls (*N* = 482) (Additional File 1: Figure S1).

Our validation study was based on the Westlake Precision Birth Cohort study (WeBirth). It is a prospective study that recruited pregnant women diagnosed with gestational diabetes mellitus (GDM) from the Hangzhou Women’s Hospital between July 2019 and October 2023 [18]. The inclusion and exclusion criteria have been described previously [18]. A total of 2001 participants were included in the cohort. In the present study, we excluded stillbirth (*N* = 1) and those without serum metabolome (*N* = 403), and finally included 1597 participants for analysis (Additional File 1: Figure S1). Anthropometric characteristics, questionnaires about medication and health behavior, and blood and fecal samples were collected in mid pregnancy (22–28 weeks’ gestation) and in late pregnancy (> 29 weeks’ gestation), respectively. The WeBirth study was approved by the Ethics Committee of Westlake University (Number: 20190701ZJS007). Written informed consent was obtained from all participants.

### Serum amino acid metabolite profiling

A liquid chromatography-electrospray ionization-tandem mass spectrometry (LC–ESI–MS/MS) system (UPLC, ExionLC AD; MS, QTRAP® 6500 + System; SCIEX, MA, USA) was used to measure serum amino acid metabolites in the THSBC and the WeBirth cohorts. Methods for the extraction of hydrophilic and hydrophobic compounds and corresponding UPLC conditions have been described in the Additional File 1.

In our analyses, we included a total of 183 amino acid metabolites in the THSBC annotated at the top 2 levels of confidence (Level 1 and Level 2; Additional File 2: Table S1), among which a total of 107 amino acid metabolites were also identified in the WeBirth cohort (Additional File 2: Table S2). These amino acid metabolites belonged to amino acids, small peptides, and amino acid derivatives. Metabolite read-outs below the limit of detection were replaced with read-outs corresponding to half of the detection limit read-out. Prior to statistical analysis, metabolite data were log transformed and converted into *z*-scores.

### Outcomes

Preterm birth was defined as delivery prior to 37 weeks of gestation. Preterm birth subtypes included spontaneous preterm birth (e.g., spontaneous preterm labor, spontaneous rupture of membranes) or medically indicated preterm birth (e.g., medical indication to plan an early labor induction or cesarean birth due to a maternal or fetal health condition, such as preeclampsia or intrauterine growth restriction) [19].

### Stool sample collection, metagenome profiling, and genotyping

The procedures of stool sample preparation and metagenome sequencing for the THSBC and WeBirth are described in the Additional File 1. Taxonomic profiling of the metagenomic samples was performed using MetaPhlAn4 (v4.0.6) [20]. Taxonomic features were filtered out if the mean relative abundance was lower than 0.01 or prevalence lower than 10%.

The genotyping procedures for the THSBC and WeBirth participants have been described elsewhere [21]. Genomic DNA was extracted from leukocytes using the TIANamp Blood DNA Kit (TIANGEN, China) followed by concentration measurement with the Qubit quantification system (Thermo Scientific, Wilmington, DE, USA). The genotyping was performed using the Illumina ASA-750 K array. Quality control of genotyping is described in the Additional File 1.

### Questionnaires and clinical data collection

Maternal age, parity, gravidity, pre-pregnancy weight and height, smoking status, drinking status, and educational levels were collected by trained staff with questionnaires in early pregnancy (THSBC) or mid pregnancy (WeBirth). We calculated the pre-pregnancy BMI as the self-reported pre-pregnancy weight (Kg) divided by squared height (m^2^). The intake frequency of major food groups (i.e., grain, fruit, vegetables, meat, egg, and dairy product) and consumption of tea and coffee during the recent 3 months preceding the questionnaire was collected in the two cohorts [18]. We applied the Chinese version of the Pregnancy Physical Activity Questionnaire [22] and the Pittsburgh Sleep Quality [23] to obtain the information on physical activity and sleep quality of participants. The collection of clinical data is described in the Additional File 1. All the blood samples were collected with overnight fasting (> 8 h), stored in ice boxes at the hospital, and then transferred to the laboratory for storage at − 80 °C within 24 h of collection.

### Statistical analysis

We used uniform manifold approximation and projection (UMAP, R package: *umap*) to visualize the dispersion of amino acid metabolites (measured using the relative standard deviation [24]) across pregnancy periods. Differences in the dispersion among the classes of amino acid metabolites were evaluated using analysis of similarity with Euclidean distances (R package: *vegan*).

In the trajectory analysis, we performed unsupervised fuzzy clustering (R package: *Mfuzz*) to cluster the trajectories of amino acid metabolites across pregnancy periods. Similar to the previous study [25], we determined the optimal number of clusters by calculating the minimum centroid distances for cluster numbers increasing from 2 to 10. Clusters with center expression data correlations greater than 0.8 were merged into one cluster. Only amino acid metabolites with memberships higher than 0.5 were retained within a cluster for subsequent analysis.

In the THSBC, we investigated the association between each standard deviation (SD) change in amino acid metabolites and gestational duration (expressed in week units) using linear mixed regression with case–control matching as random intercept, adjusted for potential confounders. The covariates included maternal age at enrollment (years), parity (nulliparous and multiparous), gravidity (nulligravid and multigravid), pre-pregnancy BMI (Kg/m^2^), educational levels (middle school or below, high school or equivalent, and college or above), gestational week at serum sampling (weeks), and batch effect (continuous), which were priori defined based on the literature [26–29]. The association between amino acid metabolites (per SD change) and preterm birth risk was investigated using conditional logistic regression adjusted for the same covariates as the above linear regression. In the WeBirth, multivariable linear regression and logistic regression were used to model the relationship of amino acid metabolites (per SD change) with gestational duration (expressed in week units) and preterm birth risk, respectively. Given that the number of preterm cases was relatively small, we combined the spontaneous preterm birth and medically indicated preterm birth categories as a commonly used strategy [30–32]. We employed the random forest method (R package: *mice*) to impute missing values in the covariates.

In our sensitivity analyses, we re-conducted the above association analysis by excluding participants with imputed covariates. We also repeated the above association analysis by imputing the metabolites with k-nearest neighbors (KNN) algorithm. We adjusted for additional covariates (in the early pregnancy data in the THSBC and the mid pregnancy data in the WeBirth) in the regression models to assess their influence on the findings: (1) smoking and drinking status (never and ever); (2) fasting blood glucose; (3) blood pressure; (4) diets and lifestyles (e.g., major food groups, consumption of tea and coffee, physical activity, and sleep quality). Given the potential inaccuracy of gravidity data, we also adjusted for only parity in our sensitivity analyses to examine the robustness of our findings. In addition, we assessed the potential effect modification by fetal sex or maternal GDM status by adding an interaction term between amino acid metabolites and sex or GDM status into the above statistical models.

Based on our identified amino acid metabolites associated with gestational duration and preterm birth, we generated an amino acid preterm prediction score (APPS) using the coefficients exacted from the above conditional logistic regression in the THSBC, and then applied the APPS to the WeBirth cohort for preterm birth prediction. The clinical model (based on age, parity, gravidity, pre-pregnancy BMI, and education [26, 27]) or the clinical and APPS model was applied to stratify patients according to their preterm birth risk. The best cut-off point was selected as the point on the curve closest to the (0, 1) point, which balanced the sensitivity and specificity of the prediction model [33]. The 95% CIs of area under the curve (AUC) were estimated with Delong’s test. Sensitivity and specificity statistics of prediction models were calculated as previously described [34]. The net reclassification index was calculated to evaluate the performance of the prediction model as the previous publication [35].

We then examined the potential determinants of the above identified amino acid metabolites and APPS. The variance in the amino acid metabolite and the APPS explained by variables belonging to each category (included demographic characteristics, clinical traits, diet and lifestyles, maternal genetics, and gut microbiota) was determined by linear models. Demographic characteristics included maternal age, parity, gravidity, pre-pregnancy BMI, and educational level. Clinical traits included fasting blood glucose, systolic blood pressure, the lipid panel, liver enzymes, and a complete blood count. Diet included major food groups (i.e., grain, fruit, vegetables, meat, egg, and dairy products). Lifestyles included smoking status, drinking status, consumption of tea and coffee, physical activity, and sleep quality. As described in our previous publication [21], we derived lead SNPs associated with the above identified amino acid metabolites for the genetic analysis using the clumping algorithm (Bonferroni-adjusted *P* < 5 × 10^−8^). The linkage disequilibrium reference panel was generated from the individual-level genotypes of East Asians in the 1 KG Phase 3 v5. The gut microbiota data were analyzed at the species level. The least absolute shrinkage and selection operator (LASSO) regression was used to model preterm birth with variables in each category (R package: glmnet). Regression coefficients were determined based on the cross-validated minimum lambda. All the variables with non-zero coefficients were selected and fitted in a linear model to compute the adjusted R-squared. We calculated the Spearman coefficients to examine the correlation between the identified amino acid metabolites as well as the APPS and the selected variables. The association between the identified amino acid metabolites and diets and lifestyles were modelled in linear regression adjusted for the same covariates as the above linear regression. All the statistical analyses were conducted with R (4.4.0).

## Results

### Cohort characteristics

The mean age (SD) was 26.9 (3.6) years for the THSBC participants, and 31.3 (3.7) years for the WeBirth participants. In the THSBC, participants with preterm delivery, compared to their counterparts with full-term delivery, tended to gain lower gestational weight (12.8 vs 14.8 kg), to be multigravida (71.4% vs 61.8%), and to be smokers (6.6% vs 5.8%) (Table 1 and Additional File 2: TableS3). A similar trend was observed in the WeBirth participants.

**Table 1:** Characteristics of study population

	**THSBC**	**Full-term**	**Preterm**	***P***	**WeBirth**	**Full-term**	**Preterm**	***P***
	**Total**				**Total**			
Participants, %	723	482	241		1597	1501	96	
Maternal age, mean (SD), y	26.9 (3.6)	26.8 (3.5)	27.2 (3.7)	< 0.001	31.3 (3.7)	31.3 (3.7)	31.7 (3.6)	< 0.001
Prepregnancy BMI, mean (SD)	21.3 (3.1)	21.3 (2.9)	21.3 (3.4)	< 0.001	22.1 (3.5)	22.1 (3.5)	22.5 (3.8)	< 0.001
Total weight gain during pregnancy, mean (SD), kg	14.1 (4.9)	14.8 (4.8)	12.8 (4.8)	< 0.001	11.1 (4.9)	11.2 (4.9)	10.0 (4.6)	< 0.001
Physical activity, mean (SD), MET-h/wk	125.9 (78.0)	130.3 (80.9)	117.1 (71.1)	< 0.001	151.2 (57.4)	152.6 (58.1)	137.9 (47.6)	< 0.001
Gestational duration, mean (SD), weeks	37.7 (2.1)	39.0 (1.0)	35.2 (1.4)	< 0.001	38.5 (1.5)	38.8 (1.0)	34.4 (2.0)	< 0.001
Parity, %				0.894				0.414
Nulliparous	428 (58.4)	284 (57.7)	144 (59.8)		1062 (66.5)	994 (66.2)	68 (70.8)	
Multiparous	295 (41.6)	198 (42.3)	97 (40.2)		535 (33.5)	507 (33.8)	28 (29.2)	
Gravidity, %				0.181				0.247
Nulligravida	252 (35.0)	183 (38.2)	69 (28.6)		730 (45.7)	691 (46.0)	39 (40.6)	
Multigravida	471 (65.0)	299 (61.8)	172 (71.4)		867 (54.3)	810 (54.0)	57 (59.4)	
Cigarette smoking status, %				0.848				0.664
Never	677 (93.9)	452 (94.2)	225 (93.4)		1529 (95.7)	1411 (94.0)	89 (92.7)	
Ever	46 (6.1)	30 (5.8)	16 (6.6)		67 (4.2)	59 (3.9)	7 (7.3)	
Alcohol drinking status, %				0.545				0.710
Never	577 (80.8)	388 (82.0)	189 (78.4)		1555 (97.4)	1432 (95.4)	94 (97.9)	
Ever	146 (19.2)	94 (18.0)	52 (21.6)		42 (2.6)	40 (2.7)	2 (2.1)	
Educational level, %				0.885				0.501
Middle school or below	127 (17.0)	87 (17.2)	40 (16.6)		177 (11.1)	164 (10.9)	13 (13.5)	
High school or equivalent	292 (39.8)	194 (39.4)	98 (40.7)		1158 (72.5)	1092 (72.8)	66 (68.8)	
College or above	304 (43.2)	201 (43.4)	103 (42.7)		233 (14.6)	216 (14.4)	17 (17.7)	

*BMI *body mass index, *MET *metabolic equivalent of task, *THSBC *Tongji-Huaxi-Shuangliu Birth Cohort, *WeBirth *Westlake Precision Birth Cohort

### Dynamics of maternal amino acid metabolites during pregnancy

**Fig. 1:**
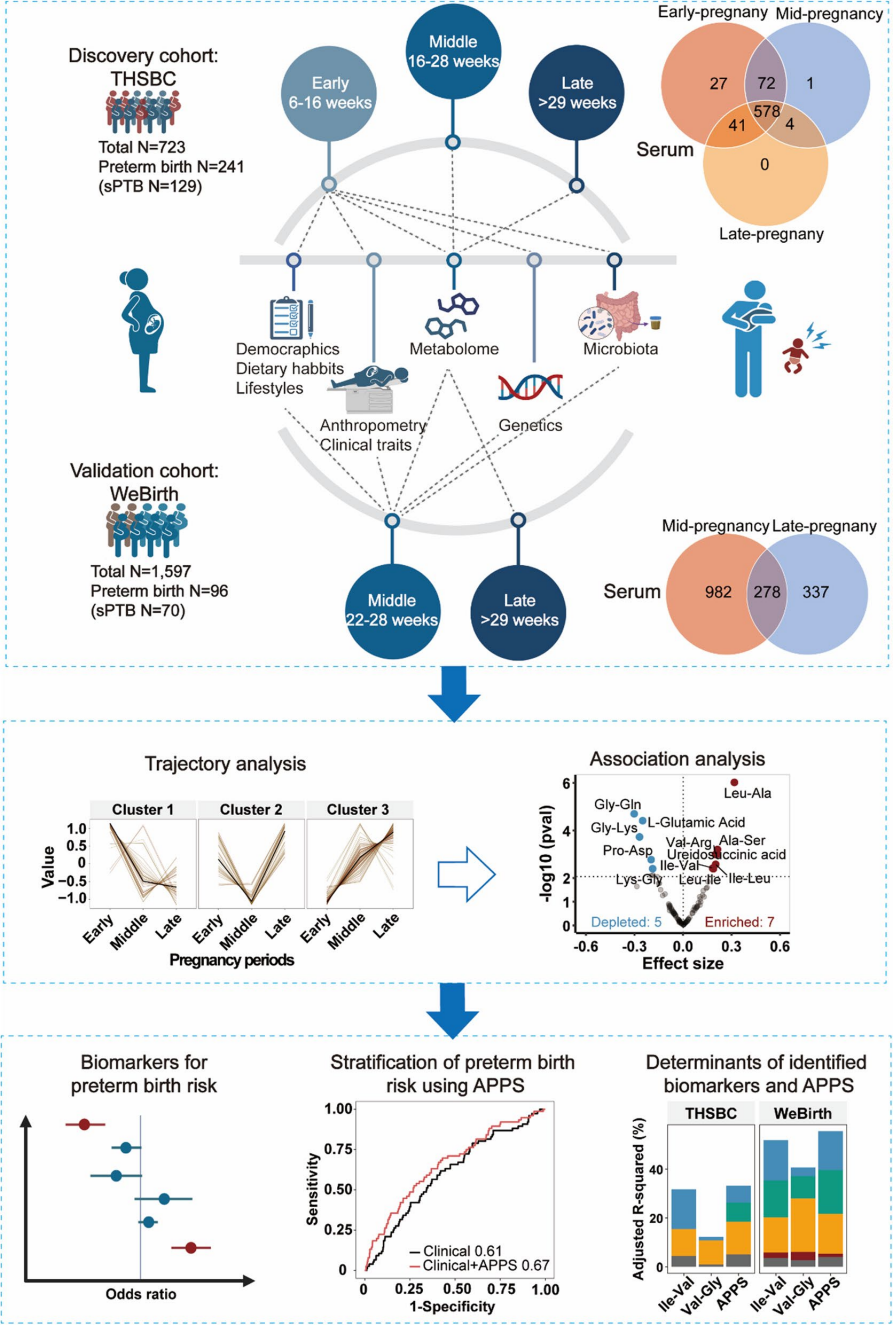
Study design. Abbreviations: APPS, amino acid preterm prediction score; sPTB, spontaneous preterm birth; THSBC, Tongji-Huaxi-Shuangliu Birth Cohort; WeBirth, Westlake Precision Birth Cohort

**Fig. 2:**
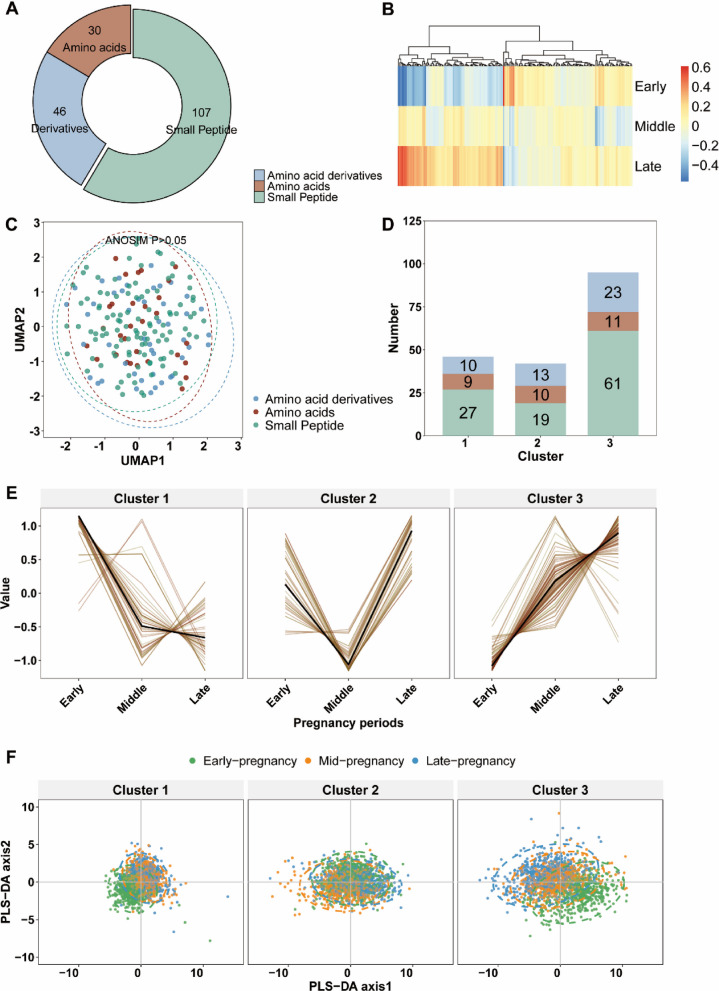
Dynamics of amino acid metabolites throughout pregnancy.Legends:** A** The number of amino acid metabolites was categorized by compound classes. **B** The heatmap showed the relative expression of amino acid metabolites. The amino acid metabolite concentrations were log-transformed followed by *z*-score standardization. **C** The UMAP plot showed the dispersion of amino acid metabolites (measured using the relative standard deviation) across pregnancy periods. **D** The trajectories of the amino acid metabolites throughout pregnancy were clustered with fuzzy clustering. The stacked bar chart showed the number of amino acid metabolites categorized by compound classes within each cluster. **E** The line chart displayed the trajectory clusters. The color varying from yellow to red indicated the increasing degree of membership. **F** PLS-DA plot visualized the amino acid metabolites of each pregnancy period in each cluster. A total of 578 participants in the THSBC were included in the trajectory analysis. Abbreviation: ANOSIM, analysis of similarity; PLS-DA, partial least squares discriminant analysis; THSBC, Tongji-Huaxi-Shuangliu Birth Cohort; UMAP, uniform manifold approximation and projection

Our study design was illustrated in Fig. 1. We depicted the dynamics of maternal amino acid metabolites in the THSBC (Fig. 2A and B), showing that the relative expression of amino acid metabolites substantially fluctuated across pregnancy periods. However, the divergent dispersion of amino acid metabolites could not be simply categorized by compound classes, given that the low-dimensional representations for 3 classes of amino acid metabolites were not distinguishable (Fig. 2C, *P* value for analysis of similarity > 0.05). In the soft clustering of the trajectories of amino acid metabolites during pregnancy, we identified 3 distinct trajectory clusters: cluster 1 with 46 metabolites declining throughout pregnancy, cluster 2 with 42 metabolites exhibiting a U-shaped trend, and cluster 3 with 95 metabolites showing a steady increase (Fig. 2D and E). We then presented the amino acid metabolites in each pregnancy period within each cluster to visualize their divergent distributions (Fig. 2F). In the PLS-DA plot, the amino acid metabolites in cluster 3 (e.g., Ile-Val) were differential across all pregnancy periods, while those in cluster 1 (e.g., γ-L-Glutamate-Cysteine) and cluster 2 (e.g., Val-Gly) were differential in early and middle pregnancy, respectively (Fig. 2F).

### Association of maternal amino acid metabolites with gestational duration and preterm birth

Only 1 amino acid metabolite was associated with gestational duration in early pregnancy, whereas 29 metabolites in mid pregnancy and 41 metabolites in late pregnancy were associated with gestational duration (Additional File 1: FigureS2). This indicated a temporal window of differential expression in amino acid metabolites for gestational duration emerging in mid pregnancy.

**Fig. 3:**
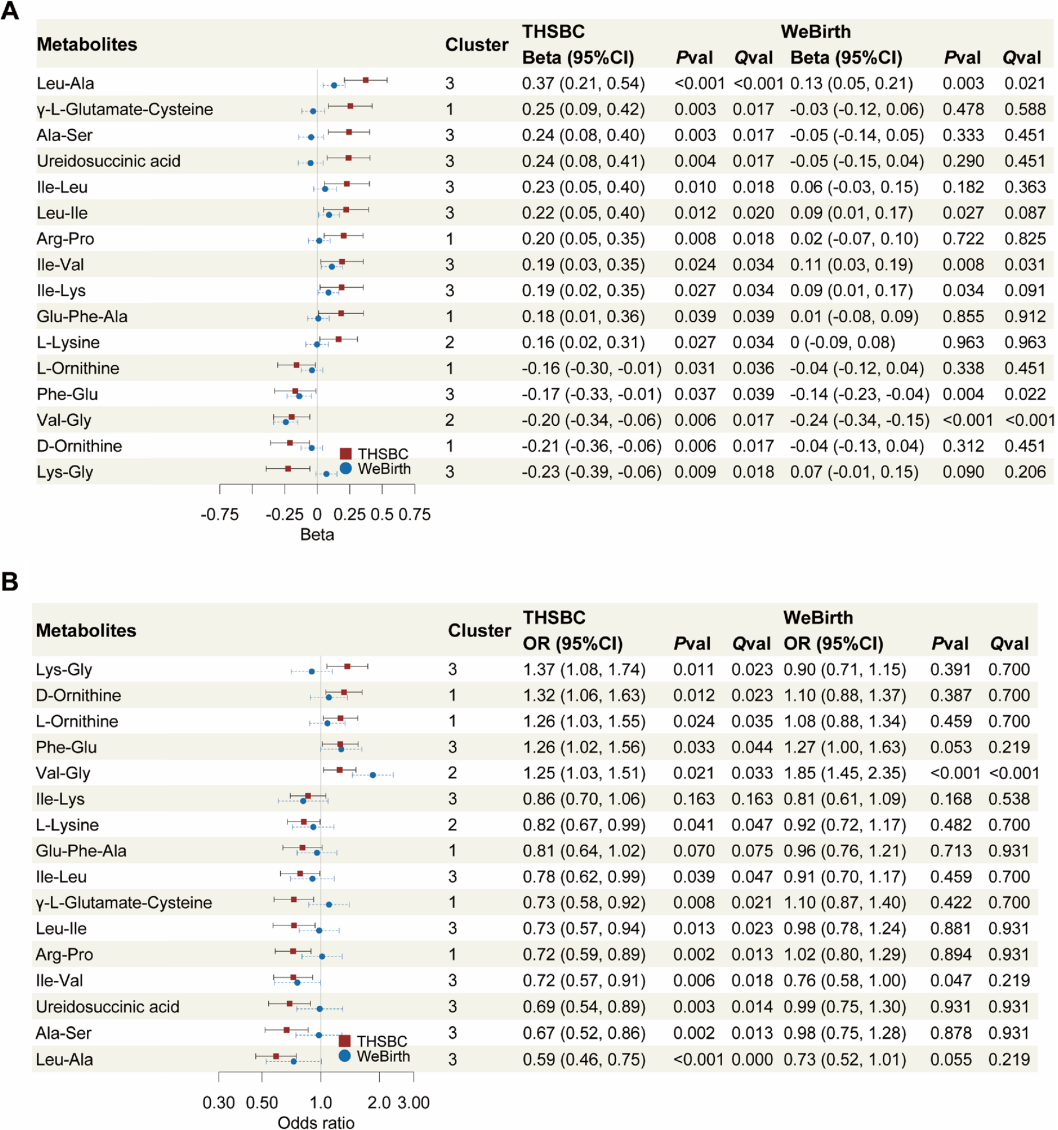
Association of maternal amino acid metabolites with gestational duration and preterm birth.Legends: Given that 29 metabolites were associated with gestational duration in mid pregnancy in the THSBC, we only showed 16 metabolites of the 29 metabolites that were identified in the WeBirth cohort. **A** The forest plot showed the relationship between amino acid metabolites in mid pregnancy and gestational duration. All the amino acid metabolites were annotated at the top 2 levels of confidence. In the THSBC (*N* = 655), linear mixed regression was fitted with case–control matching as random intercept, adjusting for maternal age, parity, gravidity, pre-pregnancy BMI, educational levels, gestational week at serum sampling, and batch effect. In the WeBirth (*N* = 1260), multivariable linear regression was fitted controlling for the above confounders. **B** The forest plot showed the relationship between amino acid metabolites in mid pregnancy and the risk of preterm birth. Conditional logistic regression and logistic regression were used to model the relationship in the THSBC and the WeBirth, respectively. Abbreviation: BMI, body mass index; NoSignifi, non-significant; *P*val, *P-*value; *Q*val, false discovery rate-adjusted *P-*value; THSBC, Tongji-Huaxi-Shuangliu Birth Cohort; WeBirth, Westlake Precision Birth Cohort

Among the 29 metabolites of mid pregnancy in the THSBC, 16 metabolites were identified in the WeBirth cohort. For the 16 metabolites, the associations between 6 small peptides in mid pregnancy and gestational duration in the THSBC could be validated in the WeBirth (*P* < 0.05, Fig. 3A). Notably, 2 small peptides were associated with the risk of preterm birth in both the THSBC and the WeBirth (*P* < 0.05, Fig. 3B). Specifically, each 1 SD increase in the serum level of Ile-Val (log transformed, hereafter the same) was associated with an increment of 0.19 (95% CI, 0.03, 0.35) week for gestational duration in the THSBC, whereas each 1 SD increase in Val-Gly was associated with 0.20 week shorter gestational duration (95% CI, − 0.34, − 0.06; Fig. 3A). In addition, each 1 SD increase in Ile-Val and Val-Gly was associated with 28% (odds ratio [OR] 0.72; 95% CI, 0.57, 0.91) lower and 25% (OR 1.25; 95% CI, 1.03, 1.51) higher risk of preterm birth in the THSBC, respectively (Fig. 3B). These associations were also observed in the WeBirth cohort.

Furthermore, Val-Gly was associated with a higher risk of spontaneous preterm birth in both THSBC (OR 1.50; 95% CI, 1.09, 2.07) and WeBirth (OR 1.60; 95% CI, 1.21, 2.12) (Additional File 1: Figure S3). In contrast, the adverse association of Val-Gly with medically indicated preterm birth was only significant in WeBirth (OR 2.90; 95% CI, 1.84, 4.57) (Additional File 1: FigureS3). We found similar results among the various sensitivity analyses (Additional File 1: Figures S4-S10) but did not observe any significant interaction for amino acid metabolites and the fetal sex or maternal GDM status (*P*_interaction_ > 0.05).

### Clinical implications of amino acid preterm prediction score (APPS)

**Fig. 4:**
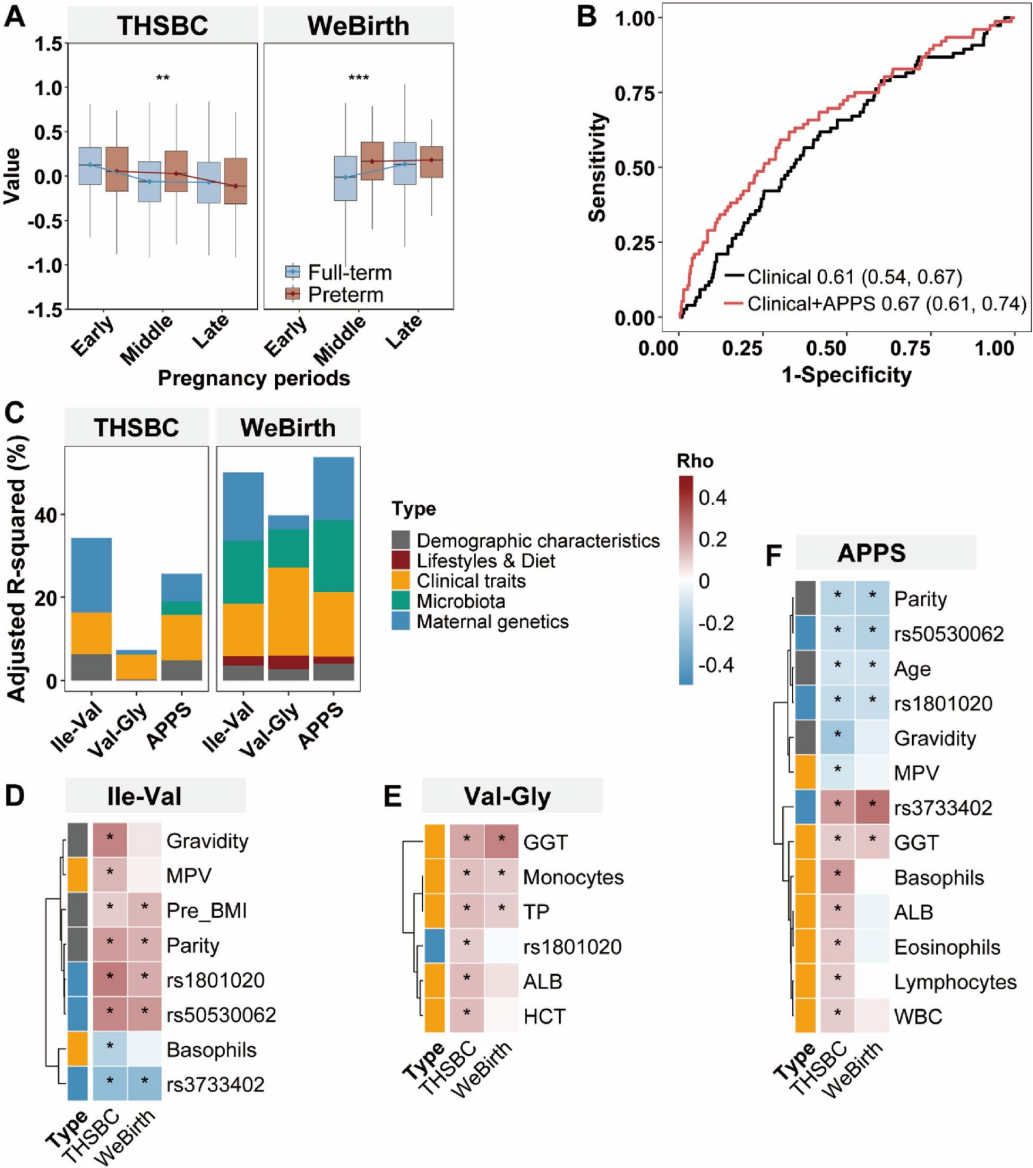
Characterization of amino acid preterm prediction score (APPS).Legends:** A** APPS was constructed based on the identified small peptides (i.e., Ile-Val and Val-Gly) (THSBC: early pregnancy *N* = 718; mid pregnancy *N* = 655; late pregnancy *N* = 623; WeBirth: mid pregnancy *N* = 1260; late pregnancy *N* = 615). The boundaries and whiskers indicated percentiles (25th and 75th) and extreme values, respectively. Asterisks represented *P*-values in Student’s *t* test (*** for < 0.001 and **** for < 0.0001). **B** The model performances in predicting preterm birth in the WeBirth (*N* = 1260). The AUC (95%CI) of clinical model and clinical plus APPS model in the THSBC was 0.59 (0.55, 0.64) and 0.63 (0.58, 0.67), respectively. **C** The explained variance of determinants for individual metabolite or the APPS in mid pregnancy was estimated in linear models (THSBC, *N* = 455; WeBirth, *N* = 518). **D** The heatmaps showed the correlation between the individual amino acid metabolites and the selected variables. Cell color represented Spearman coefficients. Asterisks indicated FDR-corrected threshold (*Q*-value < 0.25). Abbreviations: ALB, albumin; APPS, amino acid preterm prediction score; AUC, area under curve; FDR, false discovery rate; GGT, gamma-glutamyl transferase; HCT, hematocrit; LASSO, least absolute shrinkage and selection operator; MPV, mean platelet volume; OR, odds ratio; THSBC, Tongji-Huaxi-Shuangliu Birth Cohort; TP, total protein; WBC, white blood cell count; WeBirth, Westlake Precision Birth Cohort

Subsequently, we developed an amino acid preterm prediction score (APPS) based on the above identified 2 small peptides (i.e., Ile-Val and Val-Gly; coefficients were − 0.037 and − 0.27, respectively). We found that participants with preterm delivery had higher APPS than those with term delivery during the mid pregnancy in the THSBC and WeBirth (Fig. 4A, *P* < 0.001). The clinical plus APPS model had better performance in predicting preterm birth than the clinical model (AUC 0.67 [0.61, 0.74] vs 0.61 [0.54, 0.67], *P* = 0.046) (Fig. 4B and Additional File 2: Table S4). The clinical plus APPS model also improved sensitivity (10.78% vs 8.64%), but had comparable specificity to the clinical model (96.48% vs 95.95%). The net reclassification index for the clinical plus APPS model was 29.9%.

### Determinants of individual small peptides and APPS

In the present study, a total of four metabolite quantitative trait loci (i.e., rs50530062, rs1801020, rs3733402, rs502001255) were included in the genetic analysis. We found that the clinical traits explained substantive proportions of the variation in Val-Gly (ranging from 6.0% to 17.8%), while Ile-Val was primarily determined by genetics (17.8% in the THSBC and 16.5% in the WeBirth) and clinical traits (9.2% in the THSBC and 14.1% in the WeBirth) (Fig. 4C). In addition, the APPS was jointly determined by clinical factors (explained 10.2%) and genetics (explained 6.6%) in the THSBC. Interestingly, the small peptides and the APPS displayed consistent correlations with some explanatory variables in two cohorts (Fig. 4D, E, and F). For example, Ile-Val was positively correlated with the demographic characteristics (i.e., pre-pregnancy BMI and parity) and single-nucleotide polymorphisms (SNPs) (i.e., rs1801020 and rs50530062), whereas Val-Gly was positively correlated with the clinical traits (i.e., gamma-glutamyl transferase [GGT], monocytes, and total protein [TP]) (Fig. 4D and E). In contrast, neither Ile-Val nor Val-Gly was associated with diet or lifestyles (Additional File 2: Table S5).

## Discussion

This study shed light on the dynamics of maternal amino acid metabolism during pregnancy and underscored that mid pregnancy was a temporal window for amino acid perturbations linked to gestational duration. Based on the independent discovery and validation cohorts, we identified 2 small peptides that might serve as new biomarkers for gestational duration and preterm birth.

A previous study with dense sampling throughout pregnancy revealed that free amino acids exhibited similar tendencies to the trajectory clusters in our present study, including increasing, decreasing, and staying consistent [36]. For example, the plasma concentration of valine in pregnant women increased as gestation proceeded, while the concentrations of isoleucine and glycine were not significantly changed [36]. Our findings about the dynamics of amino acid metabolites (e.g., Ile-Val, Val-Gly) provided complementary knowledge for the amino acid metabolism during pregnancy. We found that more than half of maternal amino acid metabolites showed a steady increase as indicated in cluster 3, aligning with the evolving metabolic demands of pregnancy [11]. In addition, a previous study revealed significant increases in protein synthesis during the middle and late pregnancy but not the early pregnancy [37]. This temporal specificity may reflect the physiological transition from maternal tissue accretion to fetal growth acceleration during mid-gestation [38]. We found that more amino acid metabolites in mid pregnancy were associated with gestational duration relative to the early pregnancy, implying that the amino acid metabolism in mid pregnancy might have lasting impact on gestation.

Serum small peptides usually originate from dietary intake or endogenous metabolism, such as protein degradation and synthesis [39]. The identification of Ile-Val and Val-Gly as biomarkers consistently associated with gestational duration and preterm birth across cohorts is particularly interesting. Consistently, placental tissue samples from preterm birth pregnancies revealed perturbations in the glycine metabolism and biosynthesis pathways for branched-chain amino acids (e.g., isoleucine and valine) [40]. In addition, Ile-Val, known as a branched-chain amino acid derivative, stimulated glucose uptake in vitro [41]. Therefore, we speculated that Ile-Val might favor the placental nutrient transport (e.g., glucose), contributing to the gestational duration maintenance. Given that testosterone concentrations at gestation were associated with preterm delivery [42], we speculated that the positive association between Val-Gly and preterm birth might be related to the critical role of Val-Gly in testosterone homeostasis. For example, Val-Gly was recently identified as a robust biomarker for polycystic ovary syndrome [43], whose diagnosis included elevated total testosterone.

Our present study further revealed the predominant influence of the clinical parameters and genetic factors on the identified small peptides. We found that the clinical traits, especially hepatic enzyme and monocytes, were associated with Val-Gly. GGT was involved in the glutathione metabolism controlling the cellular redox homeostasis and indirectly influencing the amino acid metabolism [44]. The immune system interacted with amino acid metabolism by modulating the function of immunocytes [45]. Although the physiological status revealed by clinical traits might be an important determinant for the small peptides, more investigations are warranted to provide mechanistic insights.

Notably, 3 lead SNPs (i.e., rs1801020, rs50530062, and rs3733402) were consistently associated with Ile-Val level in the two cohorts. Our previous work and others’ work supported the *F12* locus (lead SNP: rs1801020) mapping to Ile-Val [21, 46]. The coagulation factor XII encoded by the *F12* gene was higher in GDM patients [47], but lower in women with spontaneous preterm birth [48], which was consistent with our findings. We also found that rs3733402, located at the *KLKB1* locus [46, 49], was also associated with Ile-Val level. Moreover, both rs1801020 and rs3733402 were associated with blood pressure [49]. Given that gestational hypertension is known as a component of diagnostic criteria for preeclampsia [50], our genomics findings provided some evidence for linking the identified small peptide Ile-Val and iatrogenic preterm birth.

For decades, numerous efforts have been devoted to developing biomarkers for preterm birth prediction. Among various prenatal factors, metabolomic features have been considered the best indicators for gestational age [51]. Both the top differential compounds [9] and metabolic pathways [6] could classify a large proportion of preterm cases. Notably, one previous study first developed a high-precision metabolomic clock for pregnancy and identified 5 plasma steroid hormones that could predict the approach of the delivery event [5]. However, these studies with relatively small sample sizes impeded the identification of reproducible biomarkers across cohorts. In contrast, our study revealed potentially replicable biomarkers for gestational duration and preterm birth.

There are several strengths of our present study. First, our prospective study design with repeated measurements of amino acid metabolites during pregnancy could depict the dynamics of maternal amino acid metabolism, which was rarely done in prior studies. Second, comprehensive profiling of amino acid metabolites in the present study provides a systematic view compared to previous work, which is limited to the 20 free amino acids composing proteins [14, 15]. Third, both our discovery and validation cohorts have detailed information about diets and lifestyles, microbiota, and genetics, offering multi-layer investigation for the potential determinants for maternal amino acid metabolites. Finally, our findings are validated in an independent cohort with a relatively large sample size, but also with a very different patient spectrum than the discovery cohort.

Several limitations warrant consideration. First, the predictive capacity of the APPS is tested in the WeBirth cohort which was also used to select the metabolites for the APPS, suggesting potential overfitting risk. Independent cohorts are needed to validate our findings. In addition, the AUC of our clinical plus APPS model is modest. Given that the accurate prediction of preterm birth is challenging [30], the modest AUC emphasizes that amino acid metabolic signatures cannot fully capture the multifactorial etiology of preterm birth, necessitating integration with other omics data for robust prediction. Second, the observational design precludes causal inferences, and residual confounding may persist. Third, given that our study is designed as an exploratory analysis in metabolomics, we choose a relatively loose false discovery rate (FDR)-corrected threshold (*Q*-value < 0.25) balancing the risk of false negatives against false positives. Similar approaches have been well-documented in the discovery-phase studies [52–54]. Four, the effects of amino acid metabolites on different subtypes of preterm birth are still unclear due to the limited sample size. Fifth, the association of certain amino acid metabolite (i.e., Val-Gly) with preterm birth varies greatly between the two cohorts. The dynamics of APPS score across pregnancy periods are also different in the two cohorts. These differences might be attributed to several reasons, including different population characteristics and different sampling timing (gestational age). Effectively, most of the limitations relate to the differences in patients between the validation cohort which consists of patients with GDM, and the discovery cohort which is derived from a community-based general population of pregnant women. Therefore, our findings require replication in larger cohorts, preferably in other community-based general pregnancy populations, as well as functional validation.

## Conclusions

In summary, the present study advanced our understanding of amino acid adaptations in pregnancy and suggested that circulating amino acid metabolites may serve as biomarkers for gestational duration and preterm birth.

## Supplementary Information

Additional file 1.

Additional file 2.

## Data Availability

The metagenomics data were deposited in the Genome Sequence Archive under accession number CRA014529 (for THSBC) and CRA020207 (for WeBirth) and are available at the followingURLs: [https://ngdc.cncb.ac.cn/gsa/search?searchTerm=CRA014529] (https:/ngdc.cncb.ac.cn/gsa/search?searchTerm=CRA014529 ) (for THSBC) and [https://ngdc.cncb.ac.cn/gsa/search?searchTerm=CRA020207] (https:/ngdc.cncb.ac.cn/gsa/search?searchTerm=CRA020207) (for WeBirth). The blood metabolite GWAS summary statistics are available at https://zheng.lab.westlake.edu.cn/Resource/Resource\_Preterm.htm. Other data supporting the results of the present study are available upon reasonable request from the corresponding authors (X.-F.P. or J.-S.Z.).

## Abbreviations

ALB: Albumin
ANOSIM: Analysis of similarity
APPS: Amino acid preterm prediction score
AUC: Area under curve
BMI: Body mass index
CI: Confidence interval
FDR: False discovery rate
GDM: Gestational diabetes mellitus
GGT: Gamma-glutamyl transferase
HCT: Hematocrit
KNN: K-nearest neighbors
LASSO: Least absolute shrinkage and selection operator
LC–ESI–MS/MS: Liquid chromatography-electrospray ionization-tandem mass spectrometry
MET: Metabolic equivalent of task
MPV: Mean platelet volume
OR: Odds ratio
PLS-DA: Partial least squares discriminant analysis
SD: Standard deviation
SNP: Single-nucleotide polymorphism
sPTB: Spontaneous preterm birth
THSBC: Tongji-Huaxi-Shuangliu Birth Cohort
TP: Total protein
UMAP: Uniform manifold approximation and projection
UPLC: Ultra Performance Liquid Chromatography
WBC: White blood cell count
WeBirth: Westlake Precision Birth Cohort study
