# Supplementary material for: Maternal amino acid metabolites during pregnancy and preterm birth: results from two prospective cohort studies
Source: BMC Med. 2026 Feb 18;24:170. doi: 10.1186/s12916-026-04710-5 (PMC13020148; doi:10.1186/s12916-026-04710-5)
Supplement: Supplementary file 1 — Additional file 1. [file 12916_2026_4710_MOESM1_ESM.docx]

**Supplemental Materials**

**Maternal amino acid metabolites during pregnancy and preterm birth: results from two prospective cohort studies**

Chen et al.

1. **Supplemental methods**
2. **Supplemental figures**

Figure S1 Study populations and study overviews

Legends: Study population of the THSBC (A) and WeBirth (B). Density plot in the left panel showed the distribution of sampling gestational weeks in each pregnancy period. Venn plot in the right panel showed the number of participants with amino acid metabolites profiling in each pregnancy period. Abbreviations: sPTB, spontaneous preterm birth; THSBC, Tongji-Huaxi-Shuangliu Birth Cohort; WeBirth, Westlake Precision Birth Cohort.

Figure S2 Association between amino acid metabolites and gestational duration across clusters and pregnancy periods

Legends: The volcano plots showed the association between amino acid metabolites and gestational duration in the THSBC (early-pregnancy: N=718; mid-pregnancy: N=655; late-pregnancy: N=623). Linear mixed regression was fitted with case-control matching as random intercept, adjusting for maternal age, parity, gravidity, pre-pregnancy BMI, educational levels, gestational week at serum sampling, and batch effect. The grey dots represented failing to survive FDR-corrected *P*-value threshold (*Q*-value < 0.25). The colored dots represented statistically significant metabolites identified by *Q*-value < 0.25. The horizonal line indicated the -log10(*Q*-value). Abbreviations: FDR, false discovery rate; THSBC, Tongji-Huaxi-Shuangliu Birth Cohort;

Figure S3 Association between amino acid metabolites and the risk of spontaneous preterm birth and medically indicated preterm birth

Legends: In the THSBC (N=655), conditional logistic regression was fitted for the association between amino acid metabolites and the risk of spontaneous preterm birth (A) and medically indicated preterm birth (B), adjusted for the above confounders in Figure S2. In the WeBirth (N=1,260), logistic regression was used to model the relationship. Abbreviations: OR, odds ratio; *P*val, *P-*value; *Q*val, false discovery rate-adjusted *P-*value; THSBC, Tongji-Huaxi-Shuangliu Birth Cohort; WeBirth, Westlake Precision Birth Cohort.

Figure S4 Association of amino acid metabolites with gestational duration and preterm birth in participants without imputed covariates

Legends: We reanalyzed the association of amino acid metabolites with gestational duration (A) and the risk of preterm birth (B) by excluding participants with imputed covariates. In the THSBC (N=655), linear mixed regression and conditional logistic regression were fitted for the association of amino acid metabolites with gestational duration and the risk of preterm birth, respectively. In the WeBirth (N=1,260), multivariable linear regression and logistic regression were used to model the relationship of amino acid metabolites with gestational duration and the risk of preterm birth, respectively. The same covariates of the regression in Figure S2 were adjusted for. Abbreviations: OR, odds ratio; *P*val, *P-*value; *Q*val, false discovery rate-adjusted *P-*value; THSBC, Tongji-Huaxi-Shuangliu Birth Cohort; WeBirth, Westlake Precision Birth Cohort.

Figure S5 Association of amino acid metabolites with gestational duration and preterm birth when additionally adjusting for smoking and drinking status

Legends: We reanalyzed the association of amino acid metabolites with gestational duration (A) and the risk of preterm birth (B) when additionally adjusting for smoking and drinking status. In the THSBC (N=655), linear mixed regression and conditional logistic regression were fitted for the association of amino acid metabolites with gestational duration and the risk of preterm birth, respectively. In the WeBirth (N=1,260), multivariable linear regression and logistic regression were used to model the relationship of amino acid metabolites with gestational duration and the risk of preterm birth, respectively. Smoking and drinking status plus the same covariates of the regression in Figure S2 were adjusted for. Abbreviations: OR, odds ratio; *P*val, *P-*value; *Q*val, false discovery rate-adjusted *P-*value; THSBC, Tongji-Huaxi-Shuangliu Birth Cohort; WeBirth, Westlake Precision Birth Cohort.

Figure S6 Association of amino acid metabolites with gestational duration and preterm birth when additionally adjusting for fasting blood glucose

Legends: We reanalyzed the association of amino acid metabolites with gestational duration (A) and the risk of preterm birth (B) when additionally adjusting for fasting blood glucose during mid-pregnancy. In the THSBC (N=655), linear mixed regression and conditional logistic regression were fitted for the association of amino acid metabolites with gestational duration and the risk of preterm birth, respectively. In the WeBirth (N=1,260), multivariable linear regression and logistic regression were used to model the relationship of amino acid metabolites with gestational duration and the risk of preterm birth, respectively. Fasting blood glucose plus the same covariates of the regression in Figure S2 were adjusted for. Abbreviations: OR, odds ratio; *P*val, *P-*value; *Q*val, false discovery rate-adjusted *P-*value; THSBC, Tongji-Huaxi-Shuangliu Birth Cohort; WeBirth, Westlake Precision Birth Cohort.

Figure S7 Association of amino acid metabolites with gestational duration and preterm birth when additionally adjusting for systolic blood pressure

Legends: We reanalyzed the association of amino acid metabolites with gestational duration (A) and the risk of preterm birth (B) when additionally adjusting for systolic blood pressure during mid-pregnancy. In the THSBC (N=655), linear mixed regression and conditional logistic regression were fitted for the association of amino acid metabolites with gestational duration and the risk of preterm birth, respectively. In the WeBirth (N=1,260), multivariable linear regression and logistic regression were used to model the relationship of amino acid metabolites with gestational duration and the risk of preterm birth, respectively. Systolic blood pressure plus the same covariates of the regression in Figure S2 were adjusted for. Abbreviations: OR, odds ratio; *P*val, *P-*value; *Q*val, false discovery rate-adjusted *P-*value; THSBC, Tongji-Huaxi-Shuangliu Birth Cohort; WeBirth, Westlake Precision Birth Cohort.

Figure S8 Association of amino acid metabolites with gestational duration and preterm birth when additionally adjusting for diet and lifestyles

Legends: We reanalyzed the association of amino acid metabolites with gestational duration (A) and the risk of preterm birth (B) when additionally adjusting for major food groups and lifestyles during mid-pregnancy. In the THSBC (N=655), linear mixed regression and conditional logistic regression were fitted for the association of amino acid metabolites with gestational duration and the risk of preterm birth, respectively. In the WeBirth (N=1,260), multivariable linear regression and logistic regression were used to model the relationship of amino acid metabolites with gestational duration and the risk of preterm birth, respectively. Major food groups (i.e., grain, fruit, vegetables, meat, egg, and dairy product) and consumption of tea and coffee, physical activity, and sleep quality plus the same covariates of the regression in Figure S2 were adjusted for. Abbreviations: OR, odds ratio; *P*val, *P-*value; *Q*val, false discovery rate-adjusted *P-*value; THSBC, Tongji-Huaxi-Shuangliu Birth Cohort; WeBirth, Westlake Precision Birth Cohort.

Figure S9 Association of amino acid metabolites with gestational duration and preterm birth without adjusting for gravidity

Legends: We reanalyzed the association of amino acid metabolites with gestational duration (A) and the risk of preterm birth (B) without adjusting for gravidity during mid-pregnancy. In the THSBC (N=655), linear mixed regression and conditional logistic regression were fitted for the association of amino acid metabolites with gestational duration and the risk of preterm birth, respectively. In the WeBirth (N=1,260), multivariable linear regression and logistic regression were used to model the relationship of amino acid metabolites with gestational duration and the risk of preterm birth, respectively. The same covariates of the regression in Figure S2 except gravidity were adjusted for. Abbreviations: OR, odds ratio; *P*val, *P-*value; *Q*val, false discovery rate-adjusted *P-*value; THSBC, Tongji-Huaxi-Shuangliu Birth Cohort; WeBirth, Westlake Precision Birth Cohort.

Figure S10 Association of amino acid metabolites with gestational duration and preterm birth with metabolomics imputed using k-nearest neighbors

Legends: We reanalyzed the association of amino acid metabolites with gestational duration (A) and the risk of preterm birth (B) with metabolomics imputed using k-nearest neighbors. In the THSBC (N=655), linear mixed regression and conditional logistic regression were fitted for the association of amino acid metabolites with gestational duration and the risk of preterm birth, respectively. In the WeBirth (N=1,260), multivariable linear regression and logistic regression were used to model the relationship of amino acid metabolites with gestational duration and the risk of preterm birth, respectively. The same covariates in Figure S2 were adjusted for. Abbreviations: OR, odds ratio; *P*val, *P-*value; *Q*val, false discovery rate-adjusted *P-*value; THSBC, Tongji-Huaxi-Shuangliu Birth Cohort; WeBirth, Westlake Precision Birth Cohort.

**Supplemental methods**

**Serum amino acid metabolites profiling**

The samples of the THSBC and WeBirth cohorts were analyzed in the laboratory with the same analytical methods at Wuhan Metware Biotechnology Co., Ltd. (Wuhan, China). The extraction of hydrophilic and hydrophobic compounds were conducted as the method previously described^1^. Linear ion trap (LIT) and triple quadrupole (QQQ) scans were acquired on a triple quadrupole-linear ion trap mass spectrometer (QTRAP), QTRAP® LC-MS/MS System, equipped with an electrospray ionization (ESI) Turbo Ion-Spray interface, operating in positive and negative ion mode. Instrument tuning and mass calibration were performed with 10 and 100 μmol/L polypropylene glycol solutions in QQQ and LIT modes, respectively. QQQ scans were acquired as multiple reaction monitoring (MRM) experiments with collision gas (nitrogen) set to 5 psi. Declustering potential (DP) and collision energy (CE) for individual MRM transitions was done with further DP and CE optimization. A specific set of MRM transitions were monitored for each period according to the metabolites eluted within this period. Each sample was analyzed by both positive and negative ion modes.

The mass spectrum data were processed using Analyst 1.6.3. The amino acid metabolites were identified by referencing the parent ion mass-to-charge ratio, the fragment ion mass-to-charge ratio as well as retention time of their corresponding standards in home-made Metware database and public databases. Depending on the presence of isotope internal standards or matching with the secondary mass spectrometry, we calculated scores to indicate levels of confidence in metabolite identification according to metabolomics standards initiative^2^. Consistent with our previous publication^3^, the matching score > 0.7 indicates the Level 1 confidence of metabolite characterization, while 0.5-0.7 and < 0.5 indicate the Level 2 and Level 3 confidence, respectively.

For quality control (QC), 10 μL of each sample was pooled to make up a pooled QC sample. One QC sample was injected after every 10 samples in the sequence. The coefficients of variation (CV) values of the metabolites present in all QC samples were calculated. Metabolites whose CV values were larger than 0.3 were not considered for biomarker analysis. The analytical batch size was 96. All the samples of THSBC and WeBirth cohorts were randomized within each cohort and analyzed in sequence within the same experiment. The inter-batch normalizations were performed based on QC samples. The batch effect and the run-order were adjusted for in linear models.

**Stool sample collection and metagenome profiling**

Stool samples were collected and stored in ice boxes at the hospital or home by the participants under instructions and then transferred to the hospital to store at −40 °C within 24 hours. The procedures of sample preparation have been previously described^3^. Briefly, microbial DNA was extracted with a standardized hexadecyltrimethyl-ammonium bromide (CTAB) approach^4^, followed by DNA quantification using Qubit dsDNA Assay Kit in Qubit 2.0 Fluorometer (Life Technologies, CA, USA). For DNA library preparation, a total amount of 1μg DNA per sample was used. The index codes were added to attribute sequences to each sample using NEBNext Ultra DNA Library Prep Kit (New England Biolab, MA, USA). The DNA samples were fragmented by sonication (approximately 350 bp). Then, the DNA fragments were end-polished, A-tailed, and ligated with the full-length adaptor for Illumina sequencing with further PCR amplification. Thereafter, PCR products were purified using AMPure XP system (Beckman Coulter, CA, USA), and the libraries were analyzed with Agilent2100 Bioanalyzer and quantified using real-time PCR. The clustering of the index-coded samples was performed on a cBot Cluster Generation System. Finally, sequencing was performed on the Illumina NovaSeq platform at Shanghai Personal Biotechnology Co. Ltd. (Shanghai, China) and 150 bp paired-end reads were generated.

Next, raw sequencing reads were first quality-controlled with KneadData toolkit (v0.10.0) 1) trim the reads by quality score from the 5′ and 3′ ends (quality threshold: 20); 2) removed read pairs when either read was < 50 bp, contained “N” bases, or mean quality score below 30; and 3) deduplicate the reads. Reads aligning to the human genome (H. sapiens, UCSC hg38) for decontamination via KneadData integrated with Bowtie2 (v2.4.5).

**Quality control of genotyping**

Quality control was conducted with PLINK software. The exclusion criteria included: participants with SNPs missingness > 0.05, sex discrepancy or autosomal mean heterozygosity > 3SD, participants whose first two principal components deviated > 5SD from the mean, and participants with significant genetic relatedness (IBD > 0.185). The exclusion criteria for genetic variants included: genotype call rate < 95%, deviation from the Hardy-Weinberg equilibrium with *P* value < 1×10^−5^, and minor allele frequency < 5%. Genotype imputation was conducted with the 1000 Genomes Phase 3 v5 reference panel using Minimac3. Variants with imputation accuracy RSQR > 0.3 were included in the present analysis.

**Clinical data collection**

Fasting blood glucose and 1-h and 2-h plasma glucose following 75 g oral glucose load were measured during the mid-pregnancy in the THSBC and WeBirth using a commercial glucose oxidase kit (Sichuan Maccura Biotechnology, Chengdu, China). Blood pressure (both systolic blood pressure and diastolic blood pressure) during the mid-pregnancy in the THSBC and WeBirth was measured twice with an automatic oscillometric blood pressure monitor (Omron Healthcare, Kyoto, Japan), the average value of which was calculated. Only systolic blood pressure was adjusted for in the linear regression to reduce potential multicollinearity. In addition, other clinical traits of participants were measured in serum samples collected during the early pregnancy (THSBC) or the middle pregnancy (WeBirth) at the hospital clinical laboratory. For example, the lipid panel (triglyceride, total cholesterol, high‐density lipoprotein cholesterol, and low‐density lipoprotein cholesterol) were analyzed by the Mindray BS‐200 automatic biochemistry analyzer. The liver enzymes (e.g., alanine transaminase [ALT], aspartate transaminase [AST] and gamma glutamyl transferase [GGT], alkaline phosphatase [ALP]) and indexes of hepatic function (e.g., total protein, albumin) were measured using Hitachi 7180 automatic biochemical analyzer. A complete blood count was conducted in fasting blood samples using PENTRA MS60 Automatic Blood Cell Analyzer (ABX, French).

References

1. Wu D, Shu T, Yang X, et al. Plasma metabolomic and lipidomic alterations associated with COVID-19. *Natl Sci Rev*. 2020;7(7):1157-1168. doi:10.1093/nsr/nwaa086

2. Viant MR, Kurland IJ, Jones MR, Dunn WB. How close are we to complete annotation of metabolomes? *Current Opinion in Chemical Biology*. 2017;36:64-69. doi:10.1016/j.cbpa.2017.01.001

3. Fu Y, Gou W, Wu P, et al. Landscape of the gut mycobiome dynamics during pregnancy and its relationship with host metabolism and pregnancy health. *Gut*. 2024;73(8):1302-1312. doi:10.1136/gutjnl-2024-332260

4. Arseneau JR, Steeves R, Laflamme M. Modified low-salt CTAB extraction of high-quality DNA from contaminant-rich tissues. *Mol Ecol Resour*. 2017;17(4):686-693. doi:10.1111/1755-0998.12616

**Figure S1 Study populations and study overviews**


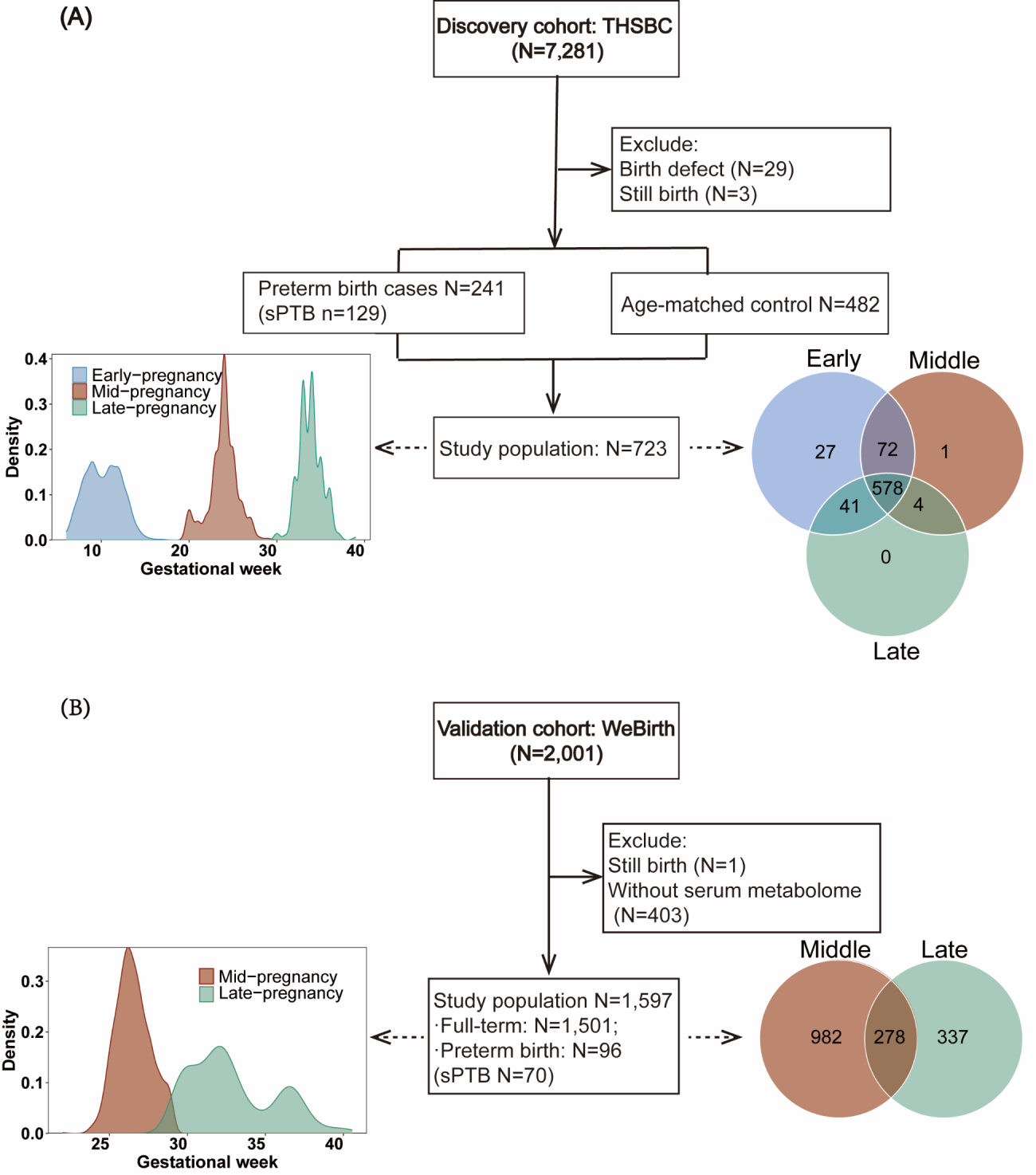


**Figure S2 Association between amino acid metabolites and gestational duration across clusters and pregnancy periods**


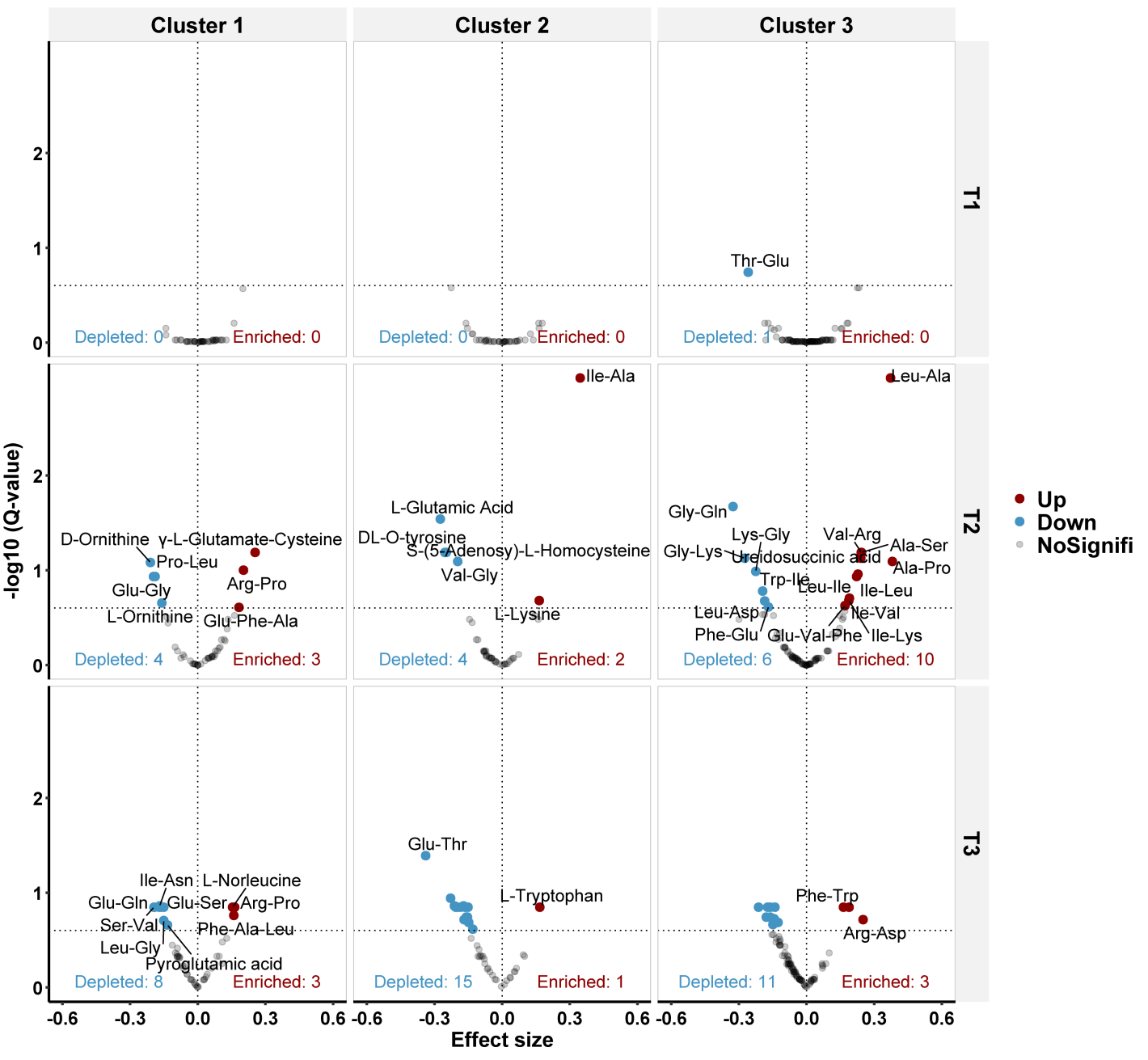


**Figure S3 Association between amino acid metabolites and the risk of spontaneous preterm birth** **and medically indicated preterm birth**


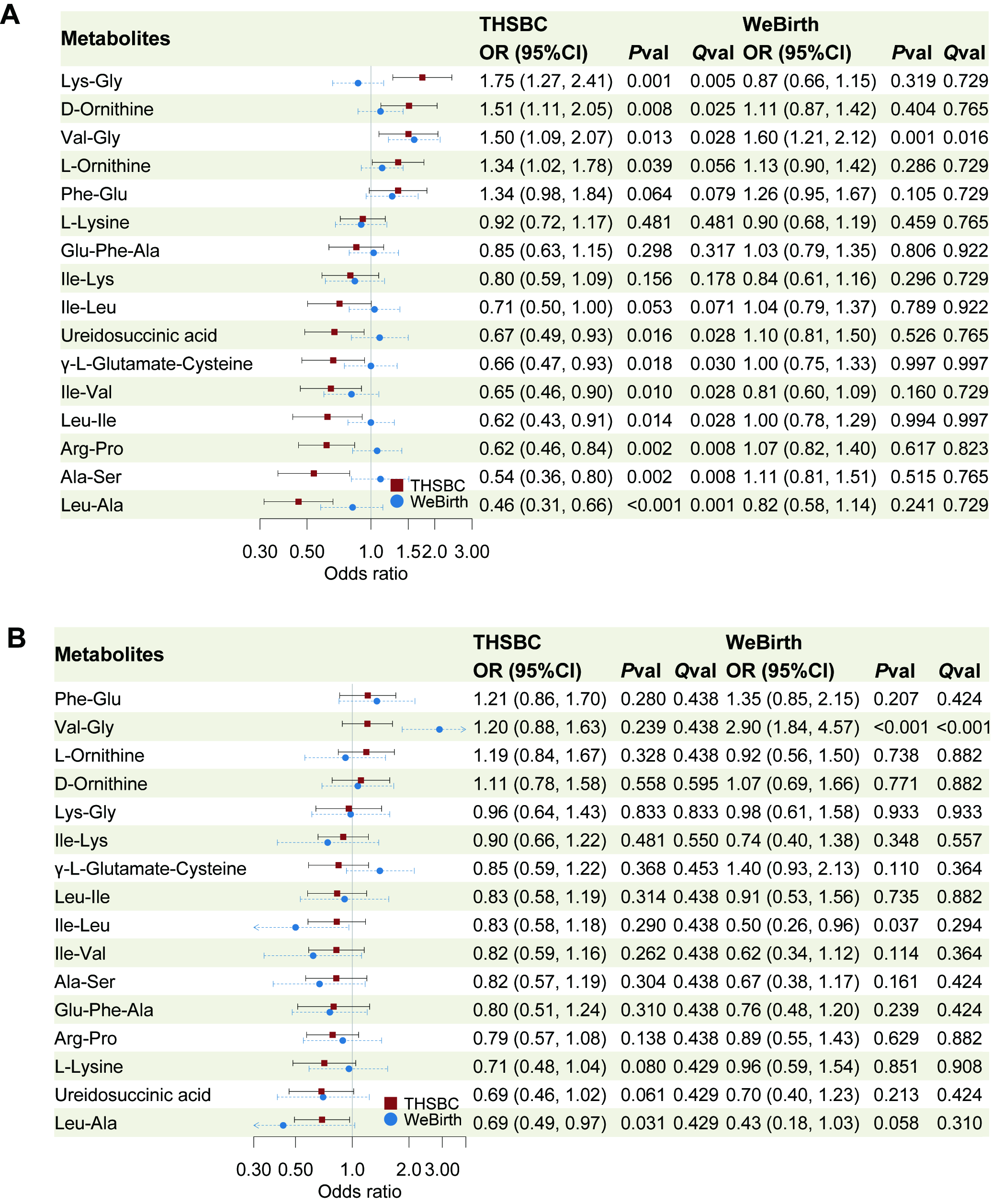


**Figure S4 Association of amino acid metabolites with gestational duration and preterm birth in** **participants without imputed covariates**


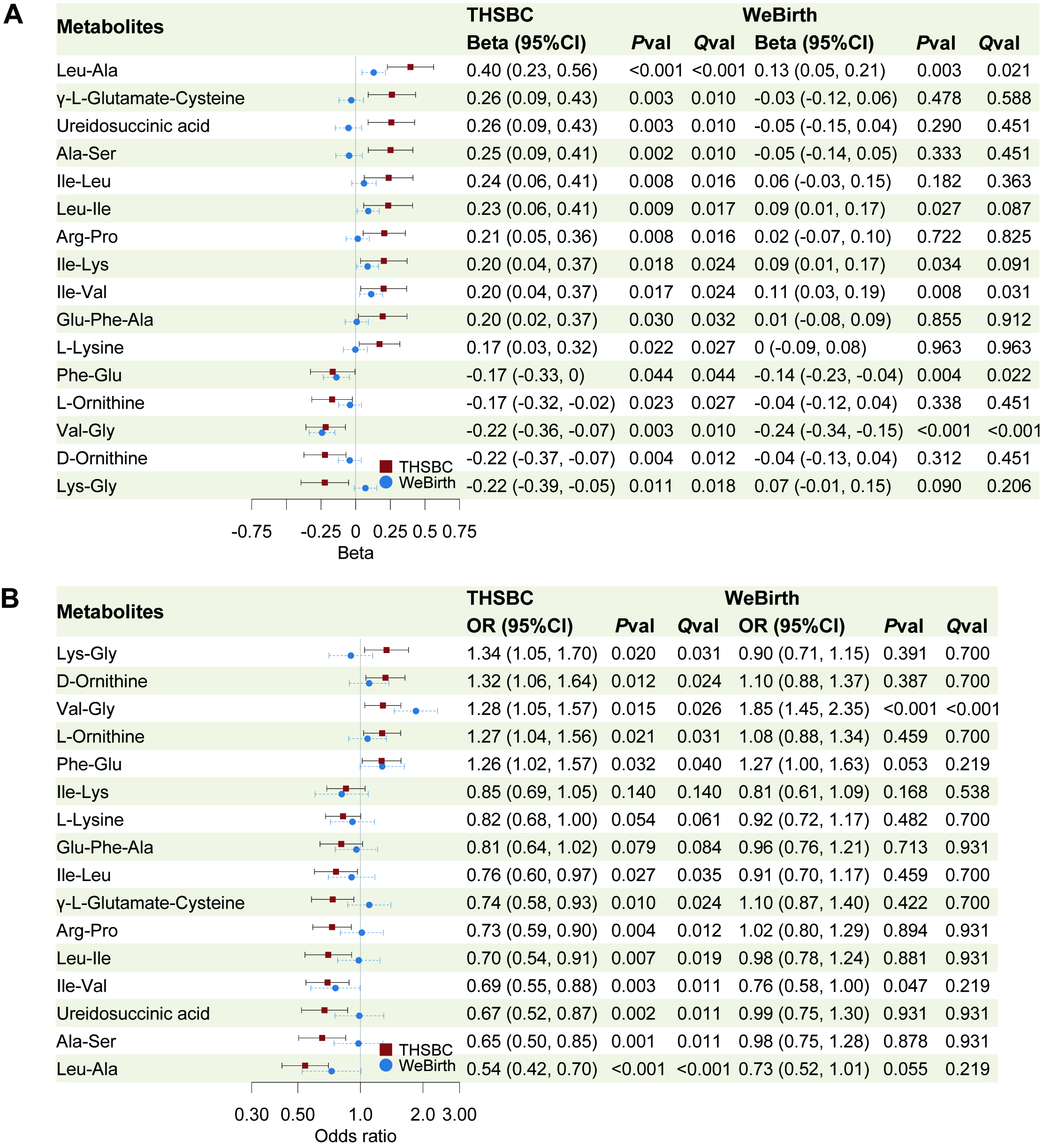


**Figure S5 Association of amino acid metabolites with gestational duration and preterm birth when additionally adjusting for smoking and drinking status**


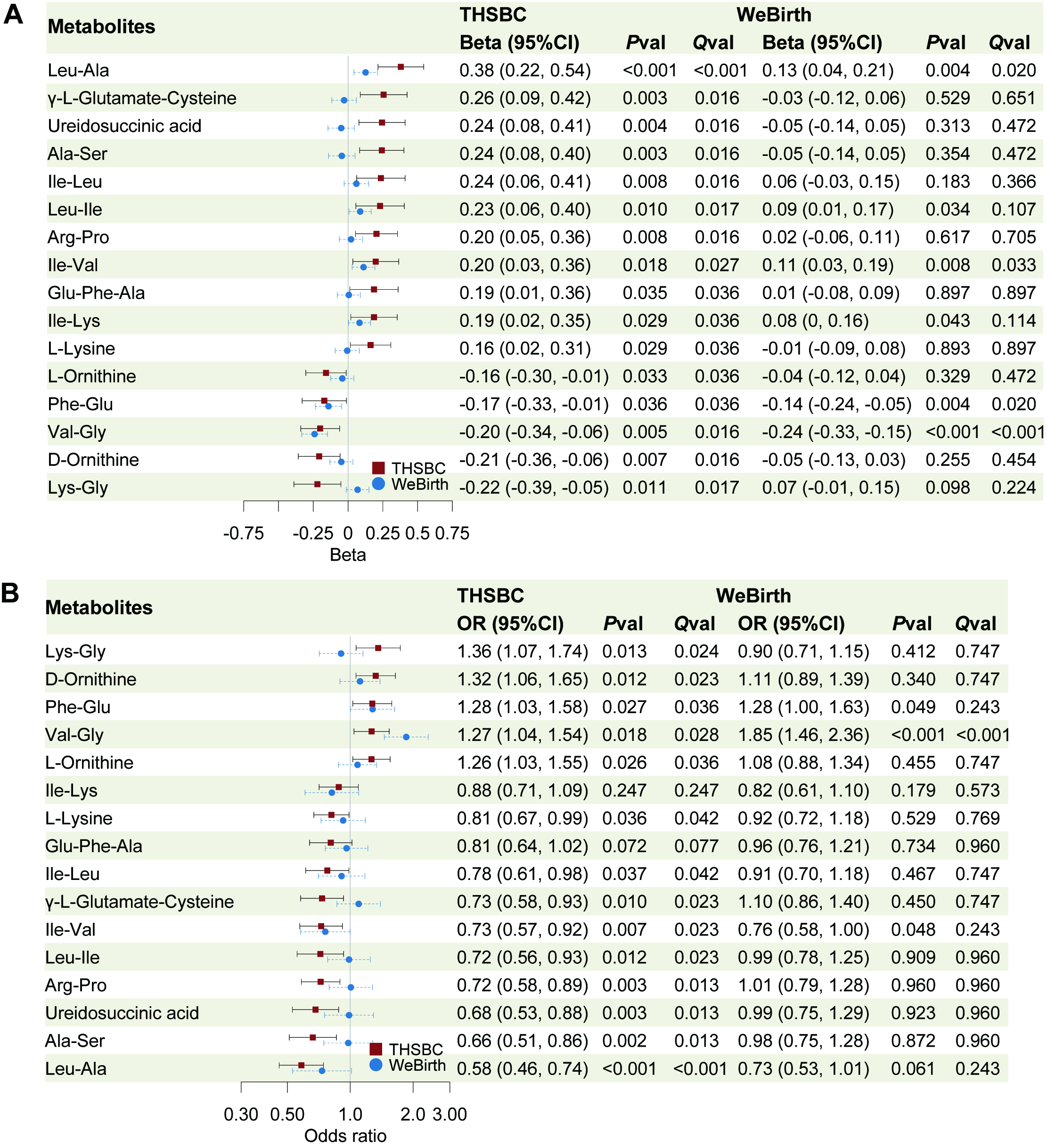


**Figure S6 Association of amino acid metabolites with gestational duration and preterm birth when additionally adjusting for fasting blood glucose**


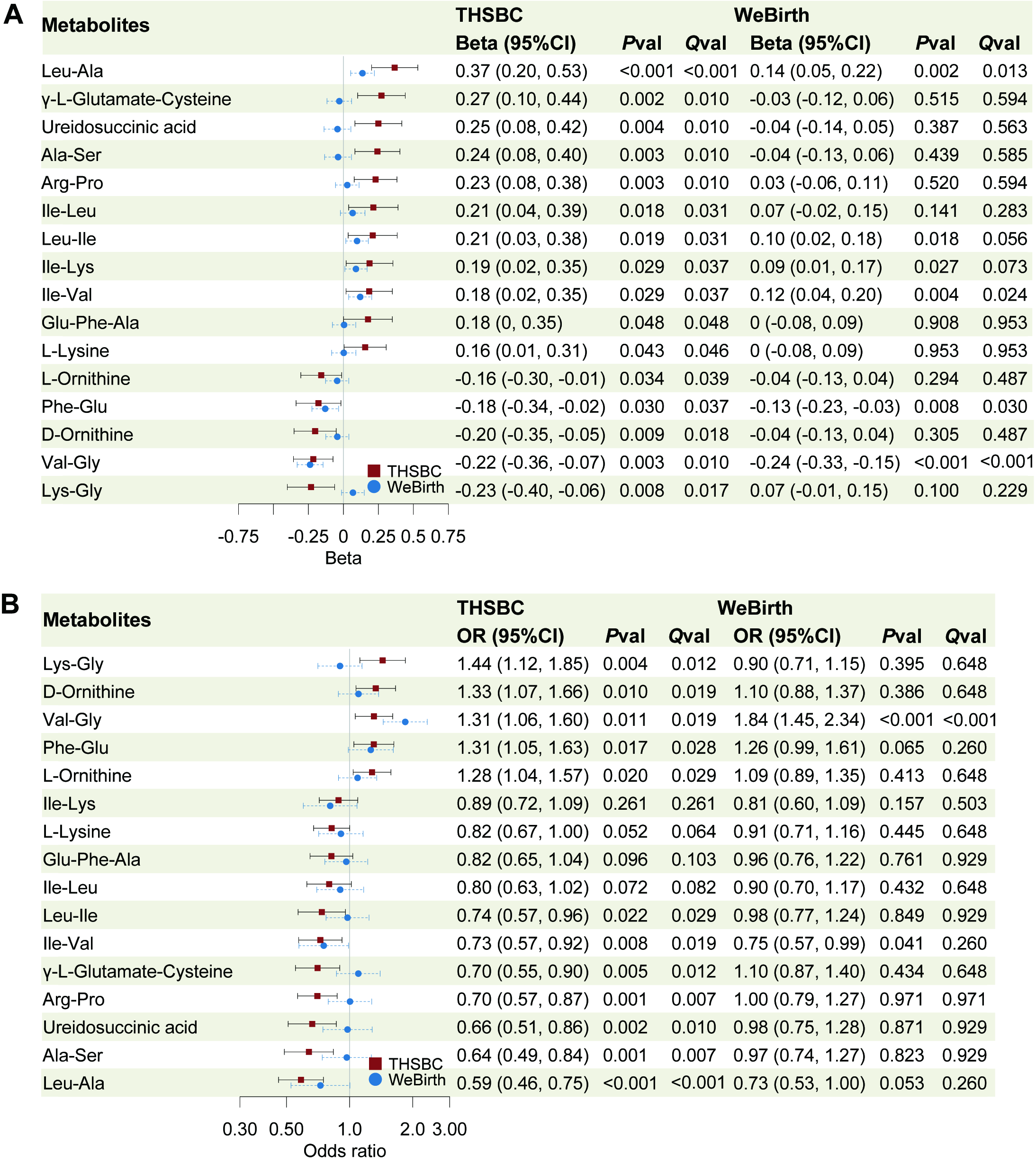


**Figure S7 Association of amino acid metabolites with gestational duration and preterm birth when additionally adjusting for systolic blood pressure**


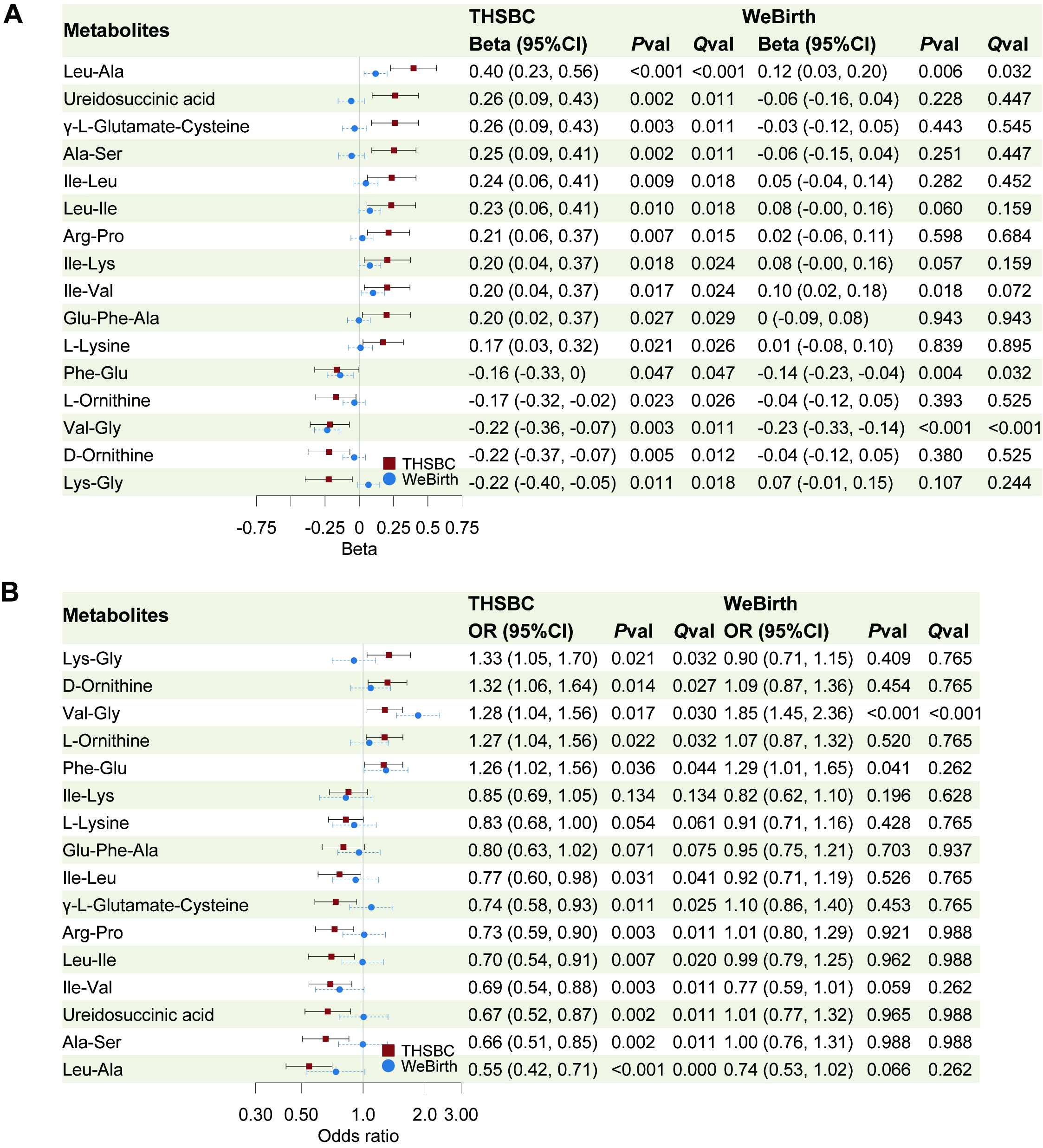


**Figure S8 Association of amino acid metabolites with gestational duration and preterm birth when additionally adjusting for diet and lifestyles**

**
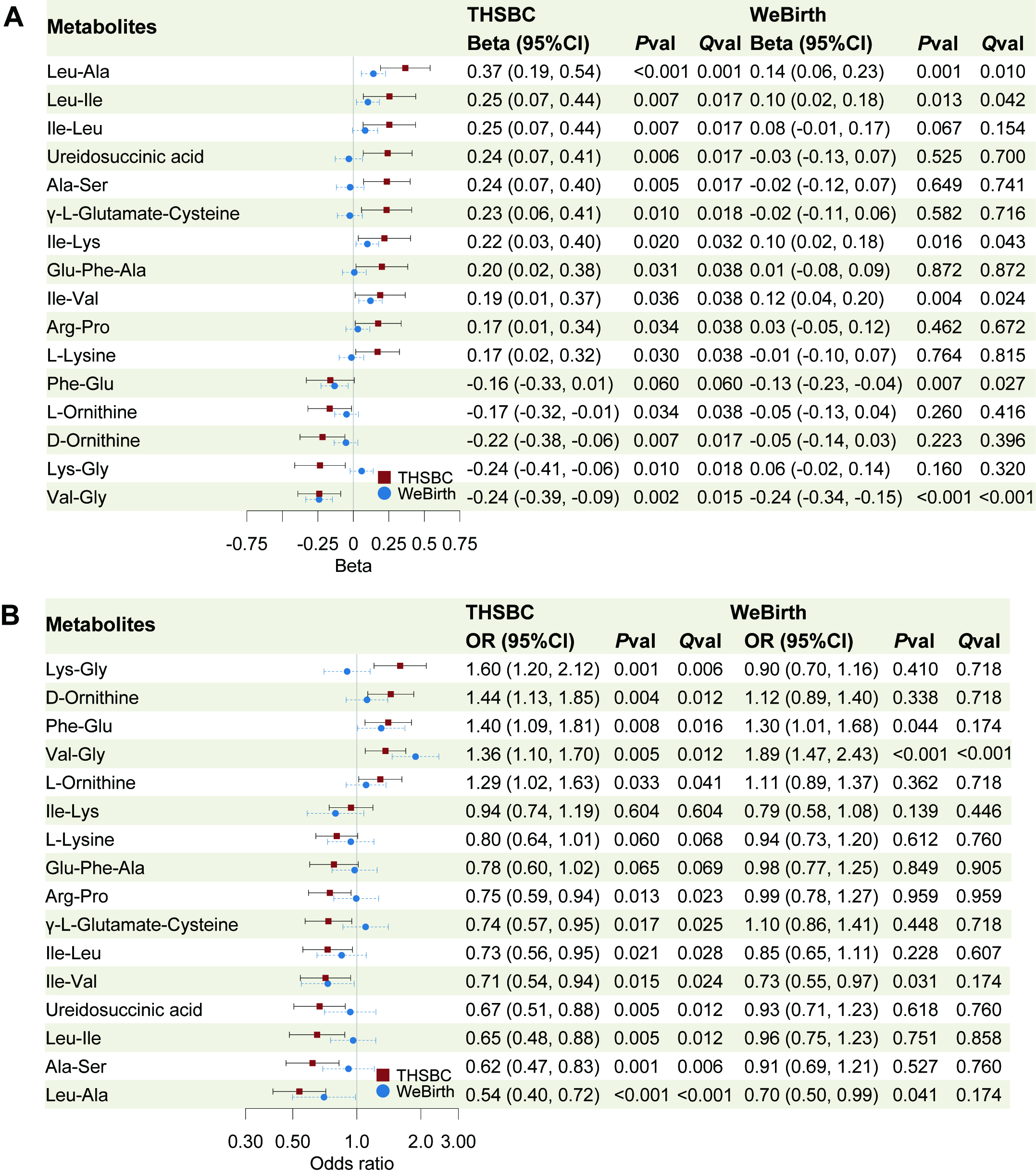
**

**Figure S9 Association of amino acid metabolites with gestational duration and preterm birth without adjusting for gravidity**

**
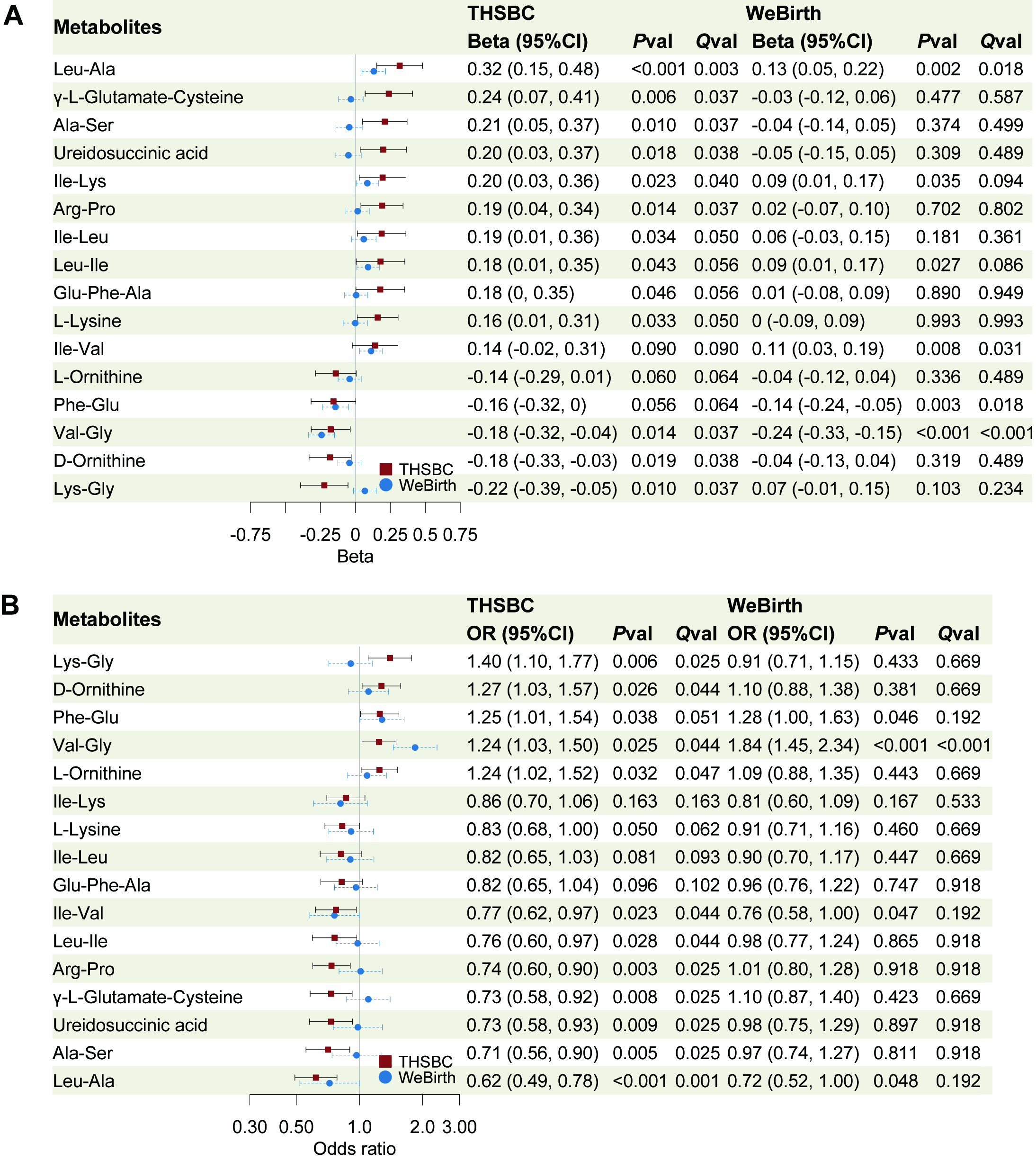
**

**Figure S10 Association of amino acid metabolites with gestational duration and preterm birth with metabolomics imputed using k-nearest neighbors**

**
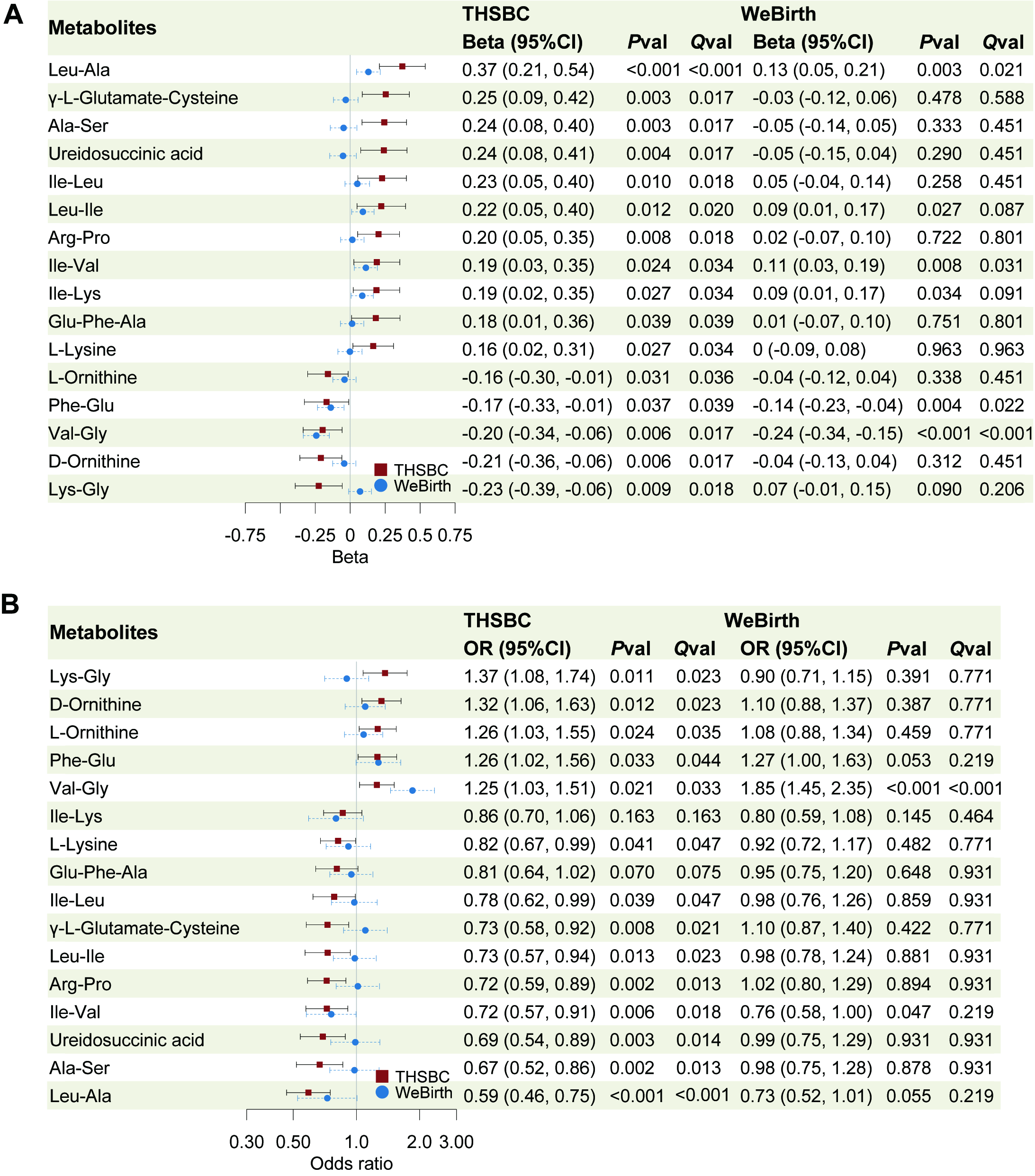
**
